# Supplementary material for: Toward an integrated approach to crop production and pollination ecology through the application of remote sensing
Source: PeerJ. 2018 Oct 19;6:e5806. doi: 10.7717/peerj.5806 (PMC6197041; doi:10.7717/peerj.5806)

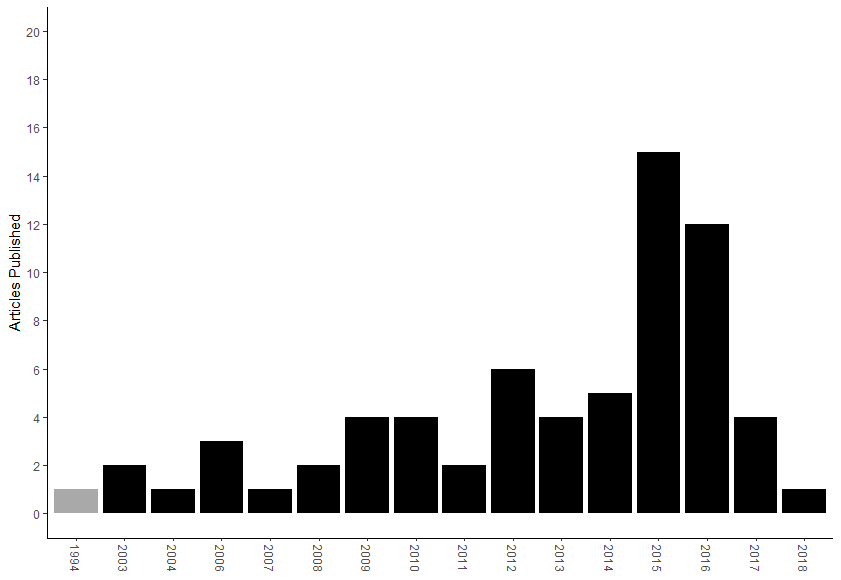


Figure 1. Range of years papers were published


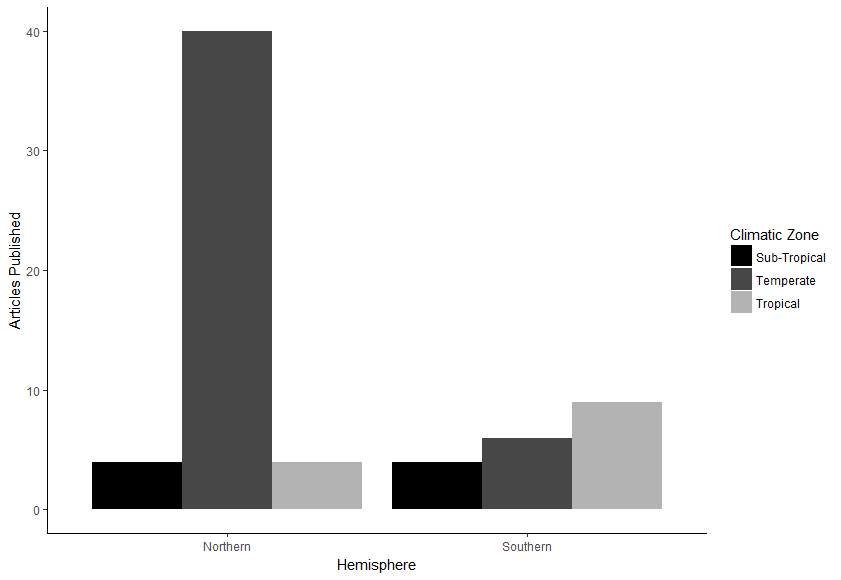


Figure 2. Biome and hemisphere


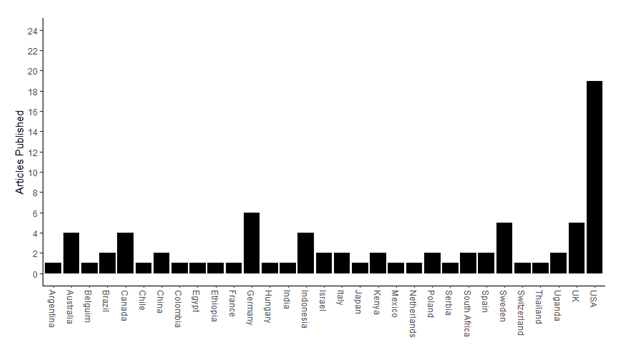


Figure 3. Country studies were conducted in.


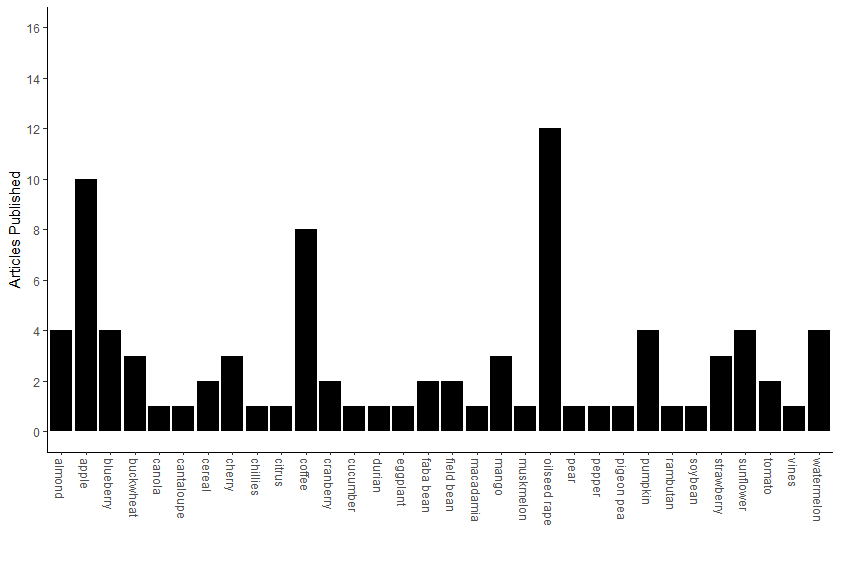


Figure 4. Crop species studies were conducted in

Table 1. Details of the 68 pollination studies that incorporated RST.


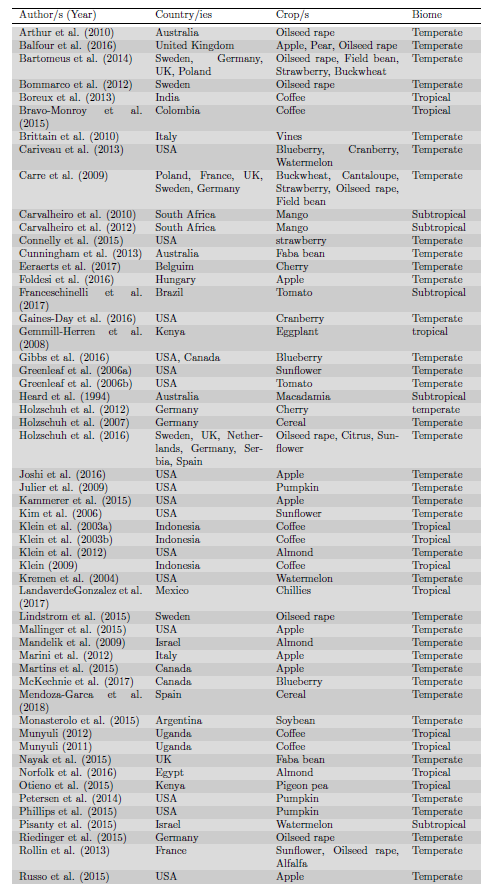


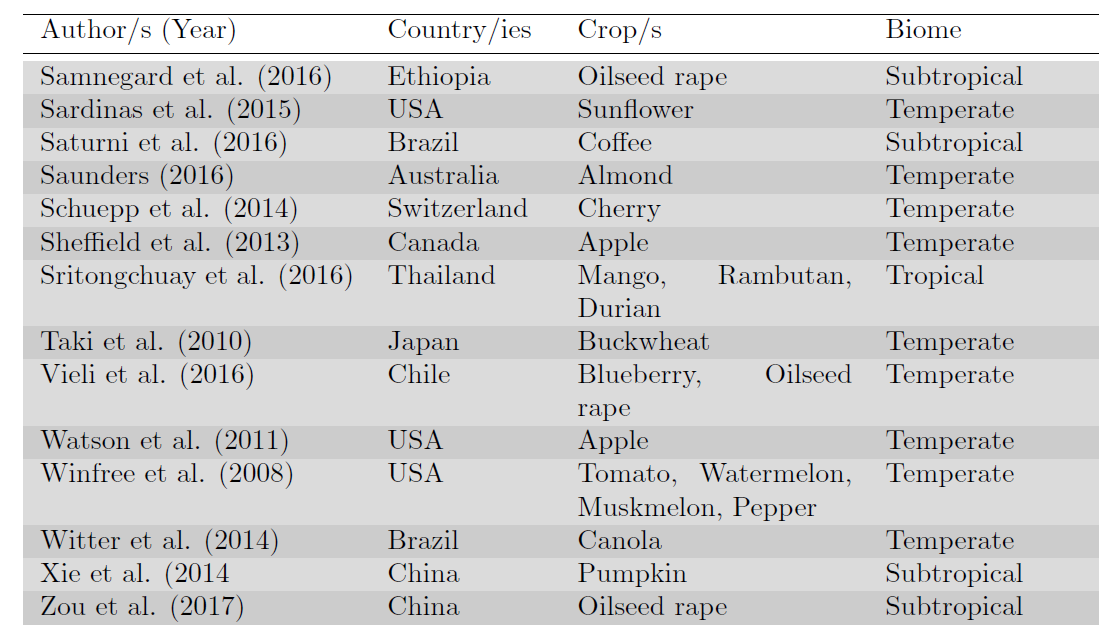

Supplement: Supplemental Information 1 [file peerj-06-5806-s001.docx]
